# A Series of Lectures on Modern Surgery, Principally from Sir Astley Cooper

**Published:** 1822-03-01

**Authors:** 


*A Series of Lectures on the most approved Principles and Practice of Modern Surgery; principally derived from the Lectures delivered by Astley Cooper, Esq. F.R.S. &c. &c. &c. at the united Hospitals of Guy and Saint Thomas, and in which will be found some of the Opinions of the most celebrated Surgeons, from the Time of Hunter, to the present Moment: interspersed with numerous Cases.* By CHARLES WILLIAM JONES. Second Edition. By CHARLES MINGAY SYDER, Surgeon. One vol. 8vo, pp. 448. London, Highly, 1821.

THERE was some doubt of the legitimacy of the first edition of this work, but the present is dedicated to Sir Astley Cooper, and acknowledged to be taken almost entirely from the lectures of this distinguished surgeon. We took an opportunity, however, of consulting Sir Astley on the appearance of this edition, and he has been kind enough to inform us that the facts in the *title page* are substantially correct; but that very many of the notes have been carelessly and even erroneously taken. Still he acknowledges that the work before us contains a broad but very imperfect outline of his lectures, as delivered in St. Thomas's Hospital. This statement will no doubt induce the majority of our surgical brethren to possess themselves of a book containing the opinions and practices of the first surgeon under (we might say *on)* the crown.*

---

* The following anecdote we have reason to believe authentic:— When Sir A. had removed a steatomatous tumour from the head of a king, His Majesty asked Mr. C***e what was the name of the tumour? Mr. C. replied, that it was called a "*steatome*"—" steatome! steatome!" observed His Majesty; "by G—d I hope it will *stay-at-home* in future, and not pay me another visit."—*Rev.*

The work consists of thirty-five chapters or lectures, embracing, in fact, almost the whole circle of surgery. It cannot be expected that we should attempt any regular analysis of an elementary volume like this; and yet, as coming from such an authority in surgery, we may well be permitted to freight a moderate proportion of our pages with matter of such high import. We shall also occasionally advert to what we consider as *misconceptions* on the part of the gentleman who took down these notes. We may remark, in limine, that the whole of the matter in this work is exceedingly interesting, though, partly from the mode of delivery and partly from the imperfect notation, it appears very unconnected, and the transitions from subject to subject, exceedingly abrupt in many instances.

We shall pass over the first lecture, with the exception of a glance at two cases related to show how wounds or accidents are modified by irritability or inirritability of constitution. A man who lived intemperately was bled by Mr. Saunders, two days after which he was taken ill. On the fifth day the arm was inflamed, and pus discharged from the wound, the pulse 120, with delirium. Opium, purgatives, and other medicines produced some mitigation, but the patient died on the ninth day. On dissection, the skin was found mortified round the arm, the cellular membrane inflamed, but the vein which had been opened was uninjured. As a contrast to this, Sir A. relates the case of a brewer's servant, who was run over by a dray, his elbow joint being opened, the bones fractured, and the artery separated from the bone. The man would not consent to amputation, so the wound was closed. It soon healed, and the man perfectly recovered. Many amputations, we are convinced, are unnecessarily performed.

"To relieve constitutional irritation, when excessive, or arising from the injury of any vital organ: 1st, take away blood, in proportion to the strength and plethoric disposition of your patient.

"2dly. Restore the secretions of the liver, kidnies, skin, and intestines.

"In children give calomel and antimonials, for nineteen diseases in twenty in them are inflammatory, and this is the best medicine to restore perspiration, &c. bathe the feet in warm water: by these means you take off the momentum of the blood, and give a healthy action to the secreting organ.

"In adults, it will be better to give calomel at night and saline purges the following morning.

"3dly. Lessen the nervous irritability by opium, combined with some sudorific.

"And, 4thly, Guide your patient's diet and regimen" 13.

The second lecture opens with one of those blunders which the student has so often fallen into in taking down the statements of the lecturer.

"The swelling in a tumour proceeds from the dilatation of the vessels *from* the effusion of coagulable lymph into the interstices of the cellular substance."

The above passage is rendered not only absurd but erroneous, from the want of "*and*" between "vessels" and "from." How could Mr. Jones imagine that the dilatation of vessels arose *from* the effusion of coagulable lymph into neighbouring cells?

Here the lecturer takes occasion to caution us against cutting on any inflamed part in amputation, since independent of the excruciating pain given, "the stump will hardly ever do well." As a general rule we believe the above is good; but naval and military surgery presents innumerable cases where we are obliged to cut through lacerated and even inflamed parts in operation, and yet the stumps do well in the majority of instances. The subject of inflammation affords much interesting matter in Sir Astley's lectures; but we can merely notice a point here and there. Alluding to the gangrene which sometimes takes place in the parts pressed upon in fevers, or where blisters have been applied, he recommends the calomel and lime water forming the black wash, as a very beneficial application, exhibiting the cinchona internally, with generous diet.

The *proximate cause* of inflammation puzzles our excellent lecturer, as it has puzzled all preceding ones. He looks upon the process itself, as generally a salutary effort of Nature to rectify some unnatural state of parts. Sir A. observes, that there does not appear to be any evident action in the vessels of an inflamed part on the blood which they receive. "This action would appear to be in the surrounding parts." "If this be an important part, (an 'important *fact*,' as the reporter absurdly has it,) it extends to the sensorium and heart itself, and causes the constitution to sympathize in severe injuries." 24. We perfectly agree with Sir Astley, that the first link in the ratio symptomatum of inflammation is an *irritation* or increase of susceptibility in the nerves of the part, which soon draws after it the affection of the sanguiferous and seriferous vessels. In the *treatment* of inflammation a great variety of admirable rules are laid down by the lecturer. We cannot but attribute to the prurient imagination of the notator such sentiments as the following:—

"Another mode of lessening inflammatory action is by perspiration, by which the edges of the crassamentum being drawn towards each other, and forming a kind of cup, drains the system of its redundancy, and drives the blood to the surface of the body." 27.

After detailing the antiphlogistic means of arresting *acute* inflammation, the lecturer observes that "when an organ has laboured long under an inflammation, so as to threaten its entire derangement or dissolution, the hydrarg. muriat. with or without Peruvian bark, according to circumstances, has been found beneficial." An instance is adduced in illustration, where a woman at Guy's Hospital had chronic inflammation of the eyes, to such an extent as to produce an opacity of the cornea. The common remedies were tried in vain; when, after taking the above medicine, the opacity of the cornea began gradually to diminish, and in a few days the inflammation subsided. The mercurial, in this instance, was combined with decoction of sarsaparilla.

In passing over a great many lectures, in every page of which we recognized the masterly observations or peculiar opinions of Sir Astley Cooper, we were struck with a remark, at page 75, where the lecturer reprobates the too common practice of opening a stump in three or four days after amputation, to see how it goes on. A stump should never be opened before the eighth or ninth day, *unless* from great pain in the part, which may lead us to suspect that the discharge cannot make its exit. On this account, intervals should be left between the adhesive straps.

In the 8th lecture on punctured wounds, we observe that Sir Astley attributes the bad effects occasionally taking place among students of anatomy from cutting or pricking their fingers, not to any morbific matter introduced into the wound, but to the irritability of the person's constitution at the time.

*Treatment.* Cut a piece of lunar caustic into a pointed shape like a point of a black lead pencil, and introduce this into the wound, and cauterize the whole internal surface of it. When this is done, very severe symptoms are prevented from coming on. If this should not have been done in the first instance, from fear of occasioning unnecessary pain, or too great confidence in themselves to be materially alarmed about such an injury, at least dilate the wound; should inflammation and pain have arisen, leeches and fomentations must be applied. The antiphlogistic regimen adopted to its full extent; calomel accompanied or succeeded by saline medicines; opium and antimony must be given at the discretion of the surgeon. From imagining that a putrid diathesis existed, many surgeons were in the habit formerly of giving wine, bark, &c. to correct it, and never allowed that the symptoms resulted from inflammation; the consequence was, that many died under this plan of treatment." 79.

Sir Astley himself suffered, some years ago, from a dissection wound. He called in the assistance of a friend, who prescribed bark and wine. He submitted to this for some time, during which all the symptoms became aggravated. He then changed the plan to antiphlogistic measures, and got well. Sir A. recommends the nitric acid, if the puncture be deep.

In the 9th lecture Sir A. makes some interesting observations on ligatures of veins. He considers this operation as very dangerous. He observes that there has not occurred one instance where the disease (varicose veins) was cured by its adoption, *unless a bandage was worn constantly afterwards;* and as this bandage alone will always relieve, if not entirely cure, which it does frequently, it is much better to use a roller and evaporating lotions, accompanied with a purgative medicine occasionally.

In division of the tendo Achillis, whether from a cutting instrument or action of the muscles, the following plan is laid down by Sir Astley.

"The heel of the patient must be raised, and the foot extended as much as possible; in order to secure the foot in this position, a piece of adhesive plaster must be bound circularly round the calf of the leg, but previous to the application of this, a broad piece must be applied from the sole of the foot, over the heel, to the calf, at its posterior part; the circular piece is then to be put on, with the intention of securing and rendering the longitudinal piece firmer in its situation; it is, therefore, to be secured above it. The slightest dressing must be applied to the wound, if there be any, and not the least pressure made upon it. However, if there exists much inflammation, leeches and fomentations must be had recourse to, to abate it, previous to the use of the plasters; great care must be taken to avoid pressure upon the ends of the tendon, otherwise it would be glued to the posterior part of the tibia and muscles, and thus prevent its motion in a great degree. The patient is to be confined to his bed four or five days, at the expiration of which time he may get up, but must immediately have a high heeled shoe put on, to prevent the rupture of the newly formed tendon, which would certainly take place, with very little exertion, if this were not done—he may be allowed to walk a little. The shoe should be one inch and a half high at the heel at least, and must be worn four or five weeks, gradually taking it down to its level." 95.

On concussion of the brain there are many good remarks in these lectures. The symptoms of this disease are not easily distinguished from sleep after excessive drinking, except by ascertaining the previous state of the patient. He appears, as it were, in a sound sleep, with a kind of delirium, and sometimes coma. His voluntary motions and senses are apparently lost, but he breathes without impediment. His pulse is rather slow, but on rousing him, or on his making the least exertion, difficulty of breathing and increased motion of the heart come on. Vomiting is generally a striking accompaniment on concussion. If the symptoms of concussion go off in a few days by rest and abstinence, there has been little damage to the brain, further than the immediate shock. The patient, even under such circumstances, should be bled, live low, and take purgative medicines. If the symptoms do not go off in five or six days, it may be concluded that inflammation has taken place, and then the most decided means must, of course, be pursued. "No patient is safe from the effects of concussion, till fourteen days after the accident." We have seen it come on a month, six weeks, and two months after the concussion.

In the treatment of concussion, it is inflammation we have to dread. We are not, of course, to draw blood *directly* after the injury, and before the powers of Nature begin to recover from the first shock. We are to wait until there be some signs of reaction, indicated by fulness and quickness of the pulse—especially of the carotid artery. Sir A. advises the temporal artery or jugular vein to be opened in preference. All the other antiphlogistic measures are also necessary. The head should be kept wet with refrigerating lotions, a large blister applied to the nape of the neck, and sinapisms to the legs.

*Compression* is thus defined in these lectures.

> "The symptoms of compression differ from those of concussion in this, that we find in the former that apoplectic stertor, which accompanies it always in a great degree, and always attended from the first instant with extreme slowness, oppressiveness, and irregularity of pulse: the loss of sense and voluntary motion is not *immediate*, as in concussion; it comes on *gradually*: the limbs are pliant, from the relaxation of the muscles: the pupils of the eye are much dilated." 148.

It is evident that pressure on the brain may take place from extravasation of fluids, blood, or serum—from depression of bone—or from formation of matter. The lecturer does not notice compression from turgescence of the vessels themselves, though this very often takes place in apoplexy, and also in cases of injury from external violence. Such an effect does not, however, come so often under the surgeon's as the physician's notice. Blood-letting, of course, is the mainspring of the treatment here as well as in concussion. If the symptoms do not yield to bleeding and other active measures, the trephine should be had recourse to, though this operation will be unavailing if the extravasation be under the dura mater or in the substance of the brain. On the Continent the trephine is almost given up, and in this country it is far less frequently applied than formerly. We should examine the head very attentively, and where pressure gives the most uneasiness there the scalpel should be applied. "After having made the opening with the scalpel, if we should *not find* the pericranium *detached* from the skull, (which will be the case if there is any fluid between the dura mater and cranium,) I do not conceive we are warranted in proceeding any farther." Sir A. makes one exception to this rule—which is, where a blow has been received on the lower part of the parietal bone, just where the artery enters. In dissection of these cases a fluid is often found extravasated between the dura matral artery and bone.

In fractures of the cranium we should deplete well, and few, comparatively, will require any surgical operation. If there be depression of bone and symptoms of extravasation, then, of course, we are authorized to apply the trephine, or such other instrument as may elevate the depression.

> "In *compound* fractures, with depression, it will be proper to trephine, whether there are any existing symptoms (of compression) or not—on the principle of preventing succeeding inflammation."

We confess that we should not be inclined to adopt strictly the above rule. We have seen very many fractures with depression, where no bad symptoms ever ensued, and who can say that an operation, in such cases, would not have done harm. As for *compound* fractures of the cranium, we consider the wound in the integuments as by no means increasing the danger—quite the contrary.

The formation of matter within the cranium is generally announced by an acute pain in the head, and cessation of discharge from the wound, which now assumes a lived and glossy appearance. The patient's countenance becomes flushed, the tunica conjunctiva injected, vomiting is commonly induced, and the patient is seized with violent shiverings. If the matter be seated exterior to the dura mater the trephine will give it vent; but yet our author has seldom seen life preserved. When the matter is under the dura mater, or in the substance of the brain, the case is nearly hopeless.

The 14th lecture is on hydrocele. Sir A. relates the case of an old man whose hydrocele was operated on at Guy's Hospital by one of the dressers. The injection passed into the cellular membrane instead of into the tunica vag. test.

and produced mortification and death. In such unfortunate instances, Sir A. recommends an incision to be made, and common water injected to lessen irritation. In young people there is danger of the injection passing into the abdomen, when the tunica vaginalis communicates with that cavity. The operation either ought not to be performed till the patient grows up; or the case should be treated, at first, as hernia, pressure being made on the mouth of the tunica vaginalis, by means of a pad, till adhesion took place. When this happens the disease may be treated by injection, as in the adult.

From the 17th lecture, we shall extract a passage containing Sir Astley's statement of the formation of aneurism.

> " The appearances that aneurism presents before the parts yield, are these: the coats of the artery are much thickened, of a yellowish hue, and opake, as if they had been subject to an inflammatory action from an injury, or disease, by which they become weakened; the thickness, with its opacity, is owing to the lymph thrown out; the part is commonly tender to the touch, and easily broken down by pressure. After this, a process of absorption commences opposite to where the opake spot is, coagulable lymph is effused, and the circulating blood pushes this before it. Hence, the sac in aneurism is formed by the fascial sheath on its outside, and by the coagulable lymph on its inside. As it increases in size the sheath becomes absorbed, the coagulable lymph soon yields, and the aneurism forms a sac with whatever is next in contact, till it reaches the skin, and through which it ultimately bursts; hence it would appear, that aneurism is *not the result of the yielding of the coats* of an artery, but *from the absorption.* If the artery be seated near a bone, it would appear that the whole circumference of the tube is not equally absorbed, and the expansion of the tumour is confined more to one side; in other cases the expansion is more uniform." 171.

In the 18th lecture Sir Astley appears to prefer puncturing the bladder above the pubis rather than through the rectum. The last operation of this kind we saw performed, was on the former printer of this Journal, Mr. Thorne, by Mr. Abernethy. A canula of elastic gum was kept above the pubis *for some months*, during which time not a drop of water came by the natural passage, and the patient was easy. At length the urine came away partly through the fistula above the pubis, and partly per urethram, which state continued till death from diseased bladder and prostate.

Upon ischuria Sir Astley makes many valuable observations. It is well known that retention of urine is generally caused by or complicated with a stricture in the urethra. In such cases Sir Astley observes that he should not think of puncturing the bladder, but cut beyond the stricture, and thus give vent to the urine

"As soon, therefore, as I should perceive the urethra to be considerably swollen and enlarged, I should direct the patient to make a violent effort to void his urine; at this very juncture, place your finger upon the perineum, and the tumour and fluctuation will be very evident, and here the incision is to commence: another similar exertion, as just stated, is to be then made, and the tumour being again distinctly felt, there will be no difficulty in proceeding with the further necessary incision, going on, as it were, mechanically. Another method, and one very easy, is to pass a staff into the urethra, as far as the stricture (for it will go no farther,) then cut down and lay it bare, then divide the stricture in the direction of the groove of the staff, carrying the knife towards the arch of the pubis." 217.

The chapters on hernia and dislocations are valuable, as may easily be imagined; but Sir Astley's regular publications on these subjects are, of course, the proper sources for information.

Speaking of gonorrhœa in the 29th lecture, the lecturer directs that, in the inflammatory stage a purgative, composed of six grains of extract of colocynth should be given every night, and two drachms of the sulphate of magnesia with a scruple of nitre every morning. The patient should drink plentifully of barley water, mucilage of gum arabic, linseed tea with nitre, &c. Low living, soda water for common beverage, open bowels, venesection if the symptoms run high, are proper. When these and various other antiphlogistic and sedative means have subdued the inflammation, the discharge may be restrained by bals. capivi, cubebs, or injections.

In the 30th lecture, Sir Astley observes that balsam copaiva is to be ranked as one of the most effectual remedies in enlarged prostate, taken in doses of twenty drops thrice daily; "but the only remedy known to give permanent relief is the hydrarg. muriat. in solution, accompanied by the sweet spirit of nitre."

On the subject of syphilis, Sir A. proves himself a decided but judicious mercurialist. He has known several instances of the fœtus in utero becoming tainted with syphilis. There is an opinion stated at page 385, that a pregnant woman cannot be cured of syphilis during utero-gestation; but whether this comes from Sir Astley Cooper or others, we are not informed. The work concludes with several lectures on fractures, scrofula, and tumours.

Sir Astley Cooper has great reason to complain of the gentleman's conduct who first published these mutilations of his lectures. We say *mutilations*, for they are so full of

*obvious* errors, that we cannot conclude otherwise than that they exhibit an exceedingly imperfect, not to say erroneous, view of Sir Astley Cooper's prælections. Another insuperable objection is this, that the annotator speaks so frequently in the nominative case, and jumbles in the same page such a variety of lecturers' names, that it is, in general, quite impossible to ascertain whose sentiments we are perusing. With the exception of these defects the work contains a great mass of very important practical matter. It is greatly to be wished that the able surgeon, whose lectures are here garbled and defaced, would employ an amanuensis to revise and print them in a form and state, worthy of the man who has so long delivered them orally to crowded audiences in the great school of which he is so distinguished an ornament.

---

